# Experience of N-acetylcysteine airway management in the successful treatment of one case of critical condition with COVID-19: A case report

**DOI:** 10.1097/MD.0000000000022577

**Published:** 2020-10-16

**Authors:** Yan Liu, Meifang Wang, Guoshi Luo, Xin Qian, Chenglin Wu, Yizhong Zhang, Biyu Chen, Elaine Lai-Han Leung, Yijun Tang

**Affiliations:** a Department of Respiratory and Critical Care Medicine, Taihe Hospital, Hubei University of Medicine, Shiyan; b State Key Laboratory of Quality Research in Chinese Medicine/Macau Institute for Applied Research in Medicine and Health, Macau University of Science and Technology, Macau (SAR), China.

**Keywords:** acetylcysteine, bronchoalveolar lavage, hypercapnia, new coronavirus pneumonia

## Abstract

**Rationale:**

The new coronavirus pneumonia Corona Virus Disease 2019 (COVID-19) has become a global pandemic. Patients with critically COVID-19 usually require invasive respiratory support, and the airway management is particularly important and the prognosis is poor.

**Patient concerns:**

A 64-year-old man with an anastomotic fistula after radical treatment of esophageal cancer and right-side encapsulated pyopneumothorax was admitted with cough and dyspnea.

**Diagnosis:**

The patient was diagnosed with novel coronavirus pneumonia and right-side encapsulated pyopneumothorax by pharyngeal swab nucleic acid test in combination with chest computed tomography (CT).

**Interventions:**

The patient was treated with antibiotics, antiviral and antibacterial medications, respiratory support, expectorant nebulization, and nutritional support. But he expressed progressive deterioration. Endotracheal intubation and mechanical ventilation were performed since the onset of the type - respiratory failure on the 13th day of admission. The patient had persistent refractory hypercapnia after mechanical ventilation. Based on the treatment mentioned above, combined with repeated bronchoalveolar lavage by using N-acetylcysteine (NAC) inhalation solution, the patients refractory hypercapnia was gradually improved.

**Outcomes:**

The patient was cured and discharged after being given the mechanical ventilation for 26 days as well as 46 days of hospitalization, currently is surviving well.

**Lessons:**

: Patients with severe conditions of novel coronavirus pneumonia often encounter bacterial infection in their later illness-stages. They may suffer respiratory failure and refractory hypercapnia that is difficult to improve due to excessive mucus secretion leading to small airway obstruction. This study provided a new insight on the proper treatment severe COVID-19 patients. The use of reasonable antibiotics and symptomatic respiratory support and other treatment, timely artificial airway and repeated bronchoalveolar NAC inhalation solution lavage, expectorant and other airway management are essential for such patients.

## 1 Introduction

COVID-19 has become a global pandemic and the number of cases and deaths continues to rise. Since the current epidemic in China has been under control, the patients in the hospital who have not yet been discharged are mainly critically conditions of which premonitorily encounter multiple comorbidities of acute respiratory distress syndrome, septic shock, intractable acidosis and multi-organ failure, with significant-high mortality rates.^[1]^ The treatment of critically ill novel coronavirus pneumonia often requires invasive respiratory support, and airway management is extremely critical. The clinical data and the evolution of the disease process of a critical novel coronavirus pneumonia patient who has been successfully treated by mechanical ventilation through tracheal intubation in the intensive care isolation ward of Taihe Hospital in Shiyan City (Hubei Medical College Affiliated Hospital) were analyzed and summarized, to provide a clinical reference for the diagnosis and treatment of critical COVID-19 patients. The study was approved by the Institutional Research Ethics Committee of Hubei Taihe Hospital. The informed consent was signed by patient for publication of this case report.

## 2 Case report

**Figure 1:**
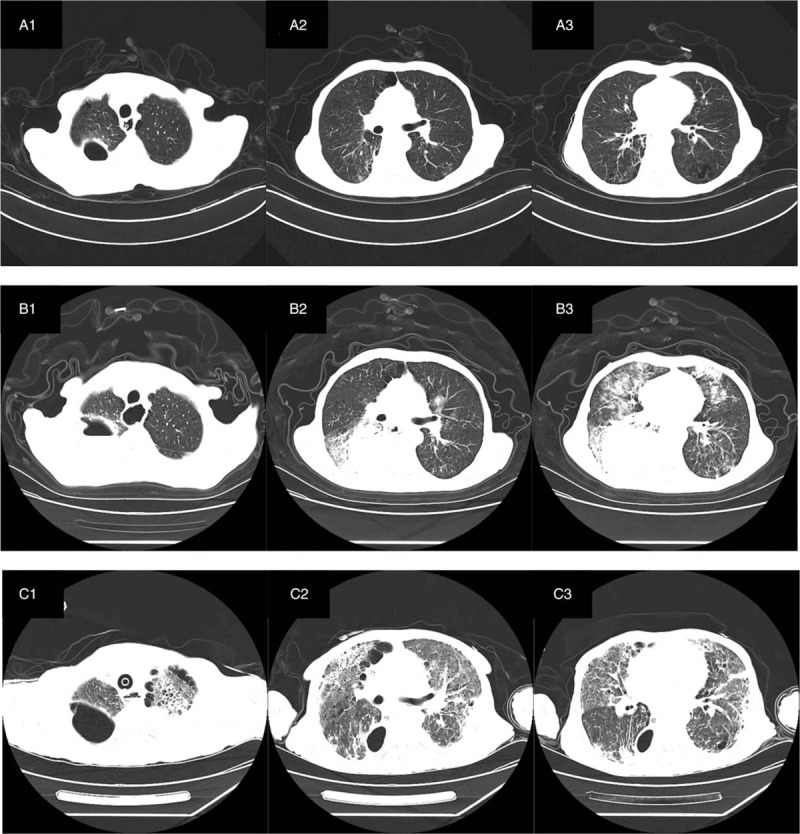
CT examination of 64 years old man diagnosed with severe COVID-19 pneumonia. (A 1–3) CT showed that there was no beak like air containing cavity in the anastomotic stoma, no communication with the right pleura, and a small amount of air accumulation and effusion in the right chest. Bilateral lungs scattered inflammation with interstitial lesions. Chronic bronchitis, emphysema. (B 1–3) CT showed that the anastomotic stoma seemed to be connected with the right pleural cavity, encapsulated pleural effusion and pneumatosis were more than before, and there were double patchy fuzzy shadows in both lungs. (C 1–3) After 30 days, CT showed that the anastomotic stoma seemed not to be connected with the right pleural cavity, encapsulated pleural effusion and pneumatosis were less than before, multiple patchy fuzzy shadows in double lungs were more absorbed than before, and the bronchial vascular bundles of both lungs were increased, disordered and interstitial thickening.

The patient, male, 64 years old, denied the trip to and from Wuhan and the history of contact with patients of a confirmed diagnosis of COVID-19 appeared a dry cough after getting up in the morning on February 16, 2020, and gradually developed shortness of breath and dyspnea. He came to the hospital that night and was admitted to the isolation ward. The patient previously underwent radical resection in the department of thoracic and cardiac surgery in Taihe hospital of Shiyan city in October 2019 due to esophageal cancer. Anastomotic fistula appeared after the operation in approximately 1 week, and the gastric tube was placed and retained for more than 2 months. On January 6, 2020, he was re-admitted to the hospital. A review of the barium meal of the digestive tract and chest CT (January 11, 2020, Fig. 1 A1-3) showed that the fistula was closed, and the gastric tube was removed, the patient was thereby discharged on January 13, 2020. Admission examination: temperature (T) 36.1°C, pulse (P) 81 bpm, respiration (R) 15 bpm, blood pressure (BP) 96/54 mm Hg, pulse oxygen saturation (SpO_2_) 98%. The patients consciousness was clear, the superficial lymph nodes were not swelling nor enlargement, the breath sounds of both lungs were weak, and that of the right lower lung was more significant. No wet and dry crackles were heard. Outpatient check-up chest CT on February 16, 2020 (Fig. 1 B1-3) revealed: anastomosis-pleural fistula after esophageal cancer, encapsulated pneumatosis and pleural effusion in the right pleural cavity, the nature of double lung infection to be determined (progression of pneumatosis, pleural effusion and local infection compared to January 11, 2020; chronic bronchitis and emphysema. Outpatient blood check routine showed: white blood cell (WBC) 8.95 g/L, netrophil (NE)% 95.2%, lymphocyte (LY): 0.4 g/L, LY%: 2.3%, red blood cell (RBC) 3.59T/L, hemoglobin (HGB) 107 g/L, platelet (PLT): 389 g/L, hypersensitive-c-reactive-protein (Hs-CRP): 197.59 mg/L, erythrocyte sedimentation rate (ESR): 60 mm/hour, procalcitonin (PCT): 0.32 ng/ml. After admission, the blood gas analysis (without oxygen) showed: pH 7.476, PCO_2_ 37 mm Hg, PO_2_ 65 mm Hg, blood lactic acid (Lac) 1.29 mmol/L. The treatment was given in single isolation, anti-infection (Cefotillar 2 g bid), relieving asthma, expectorant, nutritional support. Blood routine on the second day of admission (February 17, 2020) showed: WBC 5.62G/L, NE% 89.7%, LY: 0.44 g/L, LY%:7.8%, RBC 2.89T/L, HGB 85 g/L. His lab examination of liver function revealed albumin of 28.9 g/L with others normal; renal function, electrolytes, B-type natriuretic peptide (BNP), troponin, myoglobin, PCT were all normal. On February 17, pharyngeal swabs of novel coronavirus RNA examination was positive, novel coronavirus IgG, IgM exams were all negative. The diagnosis of novel coronavirus pneumonia was confirmed, and the patient was transferred to the novel coronary Intensive Care Isolation Unit and was given Arbidol orally, α-interferon nebulized inhaled antiviral treatment as well as traditional Chinese medicine.

The jejunal nutrient tube was recommended for the patient, but the patient's family refused. The Cefoselis was replaced with Tienam combined with moxifloxacin for anti-infection treatment on February 18. On February 19, the throat swab novel coronavirus RNA test was positive. On February 22, the Sputum culture and drug sensitivity test prompted:

1. Pseudomonas aeruginosa was found and intermediate to Piperacillin, Ceftazidine, Ticarcillin/rod acid, and was sensitive to other antibiotics;
2. Staphylococcus aurei were found and expressed multiple drug resistance.

The Patients developed wheezing aggravation on February 23, according to sputum culture results, the Moxifloxacin was replaced with Teicoplanin, and Tienam was continued to use.

The blood routine examination of February 24 showed: WBC 8.69 g/L, LY: 0.53 g/L, LY%:6.1% and the result of February 25 progressed: WBC 16.39 g/L, LY: 0.13 g/L, LY%:0.8%, which showed increased leucocytes count and progressive decline of lymphocytes. On February 25, the pharyngeal swab of the novel coronavirus RNA test was still positive. The Chest color ultrasound revealed right enveloping effusion (reduced sound transmission, not suitable for puncture drainage). Since February 25 at solstice on February 27, the patient had an acute exacerbation of intermittent wheezing and was given nasal hyperoxia therapy. Repeated examinations of BNP, troponin, PCT showed normal.

**Figure 2:**
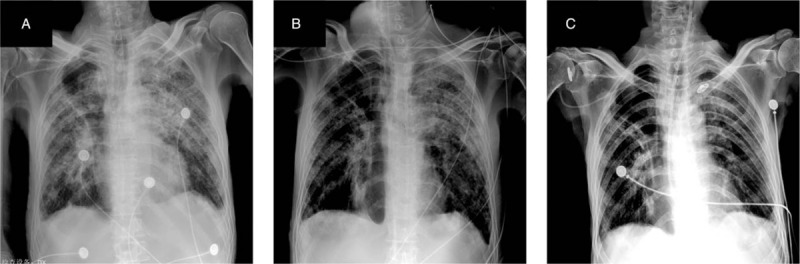
Chest X-ray examination of 64 years old man diagnosed with severe COVID-19 pneumonia. (A) On February 28, after tracheal intubation, chest X-ray showed interstitial inflammation of both lungs and a small amount of pleural effusion on both sides. (B) 7 days after tracheal intubation, chest X-ray examination showed multiple patchy increased density shadows in both lungs, which were more than before, and a small amount of pleural effusion on both sides was roughly the same as before. (C) Chest X-ray examination on March 28 before discharge showed multiple patches and strip like density shadows in double lungs, which were significantly improved than before. The right chest contains an air sac cavity.

On February 28, the patients respiratory rate was around 35 times/minutes, pulse was around 130 times/minutes, and blood gas analysis reported: pH: 7.361, pCO_2_: 66.3 mm Hg, hence the patient was given non-invasive ventilator ventilation, at the same time, the blood gas analysis was performed intermittently which revealed the pCO_2_ had not decreased significantly. The patient became unconscious after given noninvasive ventilator ventilation for about 4 hours. Therefore, tracheal intubation, as well as mechanical ventilation (V/AC mode, Vt 380 ml, positive end-expiratory pressure (PEEP) 6cmH_2_O, fraction of inspiration O_2_ (FiO_2_) 95%), was performed. At the beginning of the mechanical ventilation treatment, the ventilator alarmed of airway hypertension (up to 47 cmH_2_O) repeatedly and the blood gas report was reviewed again: pH: 7.240, pCO_2_: 110.3 mm Hg. Consequently, the emergency bedside bronchoscopy under tertiary protection was performed and showed yellowish-white purulent secretions within the trachea, left and right bronchi. Mobilized chest X-rays examination revealed that the right side of the enveloping purulent lumen disappeared, and the double lung infection was aggravated (Fig. 2A). After the bronchoscopic saline lavage to clear airway secretions, the blood gas analysis reported (Vt 350 ml, PEEP 6cmH2O, FiO2 80%): partial pressure of carbon dioxide in artery (PaCO_2_) 86.6 mm Hg, which indicated the PaCO2 was slightly improved but still in high level.

From March 1 to March 11, bedside bronchoscopy NAC inhalation solution lavage to clean the airway was performed at intervals of 1 day or 2 days: after each negative pressure suction of airway secretion was cleared under bronchoscopy, 10 to 15 g of NAC solution was infused into each bronchus alternately in the left and right bronchial tubes, retained for 2 to 3 minutes, and the airway secretion was cleared again by negative pressure suction. During the disease, the blood gas analysis was dynamically reviewed, and the patients PaCO_2_ gradually decreased to the normal range. On March 3, XueBiJing was administrated with a 100 ml bid combined with ornidazole 0.5 g qd. On March 4, the chest X-ray examination showed that the lung infection had progressed. On March 5, methylprednisolone 40 mg qd was given, and at the same time, the family of the patient finally agreed to place the jejunal nutrition tube and began enteral combined with parenteral joint nutritional support.

The sputum and bronchoscopic lavage fluid of the novel crown virus RNA tests were negative on February 28, March 1, and March 5. Both sputum culture and bronchoscope lavage fluid culture on March 5 indicated the detection of Pseudomonas aeruginosa. Re-examination of T lymphocyte subsets on March 7 reported: total T cells (CD3 +) 171 /μl, helper T cells (CD4 +)% 26.47%, CD4 + / CD8 + 0.75, which indicated reduced universally, cytotoxic T cells (CD8 +)% 35.44%, appeared with increasing; re-examination of the chest X-ray on March 7 showed that the lung infection was significantly improved (Fig. 2B). The patient's blood gas analysis was reviewed on March 8 (synchronized intermittent mandatory ventilation (SIMV) + pressure support ventilation (PSV), PS 12 cm2O, f 18 times/minutes, PEEP 6cmH_2_O, FiO_2_ 50%) and reported: PaCO_2_ 53.9 mm Hg, which was significantly decreased, the descending antibiotic ladder was changed to Sulperazone combined with levofloxacin. Re-examination of blood gas analysis reported pCO_2_ of 46.3 mm Hg on March 12, the patients hypercapnia was significantly improved, and methylprednisolone was reduced to 20 mg qd. On March 17 (31st day of admission, 18 days after endotracheal intubation), the patient began to disengage from the ventilator intermittently. The endotracheal intubation catheter was given an artificial nose intermittently, and the spontaneous breathing test was performed intermittently. Re-examination of chest CT on March 18 (Fig. 1 C1–3) revealed that the anastomotic pleural fistula after esophageal cancer surgery, the pleural effusion of the right chest cavity decreased compared with previous examinations; the infectious lesions of both lungs were partially absorbed, consequently the methylprednisolone application was terminated. On March 22, the patient again performed bedside bronchoscopy to clear the airway secretions and successfully removed the tracheal intubation, given nasal high-flow oxygen therapy and continued to anti-infection, airway management, nutritional support, immunity enhancement, and respiratory function exercises treatment. On March 28, the chest radiograph was reviewed: the interstitial inflammatory lesions of both lungs were significantly improved (Fig. 2C). The patient's inflammation index decreased from Hs-CRP: 197.59 mg/L at admission to 22.41 mg/L and IL-6 decreased from the initial 94.00 pg/ml to 8.17 pg/ml. On April 1, the patient was cured and discharged with a jejunal nutrition tube. In the entire diagnosis and treatment process, patients received a total of 1700 ml of plasma, including 600 ml of plasma of convalescent patients with novel coronavirus pneumonia, 10 U of red blood cells, 300 g of albumin, and 50 g of static C, and 16 mg of thymalfasin during the recovery period.

## 3 Discussion and conclusions

In this case, the patient was admitted to the hospital with cough and respiratory distress, although denying a clear history of epidemic exposure, the patient described a family of 3 members (grandmother, daughter and granddaughter) of a neighboring family about 300 m apart from him was diagnosed with novel coronavirus pneumonia 10 days before his onset, in which the neighbor's daughter returned from Wuhan in late January, the patient lived with his daughter and son-in-law, and his family never had a confirmed case. The analysis of the etiology may be related to the underlying disease, weak resistance. The patient developed exacerbated dyspnea on the 7th day of the onset, and mechanical ventilation by tracheal intubation was performed 13 days after the occurrence. According to the Seventh Trial Version of the Novel Coronavirus Pneumonia Treatment Protocol, the patient was diagnosed with a clear critical category for novel coronavirus pneumonia typing. Studies have shown that,^[2–5]^ the risk of infection is higher in elderly patients with combined pulmonary and cardiac underlying diseases and diabetes mellitus, and the mortality rate is higher in older men with combined underlying diseases; in this case, the patient with combined chronic bronchopneumonia and emphysema, and also had anastomotic pleural fistula after radical treatment of esophageal cancer, right-sided encapsulated pyopneumothorax, and malnutrition, with risk factors for serious illness, which significantly increased the difficulty of treatment. The Patient showed a progressive decrease in blood lymphocyte count after admission, with a significant increase in IL-6 and a significant decrease in CD3+, CD4+/CD8+, indicating high levels of inflammation, severe disease severity, and severely impaired immune function, consistent with the available studies.^[6–8]^

In this case, as the patients condition developed and progressed, the patients diagnosis, which was based on imaging, blood gas analysis and other tests, had been considered secondary bronchial fistula, bacterial lung infection, and acute respiratory distress syndrome (ARDS): persistent carbon dioxide retention and hypercarbonemia after mechanical ventilation by endotracheal intubation. However, the clearance of airway secretions does not improve hypercarbonemia well, therefore, in conjunction with a review of the literature to consider small airway mucosal embolism secondary to ARDS due to novel coronavirus infection and secondary bacterial infection, studies have shown that most patients with novel coronavirus pneumonia encountered the problem of sputum/mucus excess. The autopsy report of the death of a patient with novel coronavirus pneumonia showed that:^[9–11]^ the airway of the patient with novel coronavirus pneumonia showed a large amount of mucus secretion, and the section showed a large amount of mucus secretion spilled from the alveoli; the histological findings showed that:^[10]^ the patient had diffuse alveolar injury of both lungs with cellular fibrous mucinous exudation, all of which were visible interstitial lymphocyte-based mononuclear cell inflammatory infiltration.

As a mucolytic agent, NAC not only has the effect of directly dissolving mucus expectorant but also has the effect of anti-inflammatory, antioxidant, increasing the secretion and activity of active substances in the lung.^[12–15]^ A large number of free radicals are produced in ARDS patients. Endogenous antioxidant substances may reduce the damage to target cells, lung interstitial fibrosis. Recommendations for Nebulized Inhalation Therapy for Patients with Novel Coronavirus Pneumonia^[16]^ also recommend NAC inhalation therapy. Some scholars have applied NAC nebulized inhalation solution diluted as bronchoscopic lavage solution for patients with severe pneumonia with good results. Besides, N-acetylcysteine injection is used for the treatment of liver failure at an intravenous dose of 8 g/day for 45 days. The treatment protocol was based on the above analysis of the condition and the available literature references. In this case, the patient underwent repeated bedside bronchoscopic administration of large doses (10–15 g) of NAC nebulized inhalation solution for airway irrigation. The gradual improvement of the patient's hypercarbonemia and the apparent improvement of the absorption of the lung infection of the patient sufficiently indicated that repeated airway management by bronchoscopic NAC inhalation solution lavage might play a decisive role in the successful treatment. This case is the first report of the use of high-dose NAC nebulized inhalation liquid as lavage fluid in the airway. Although there is a certain literature base, its effect on local airway mucosa, lung absorption, and effects on other human organs after absorption is yet to be demonstrated. In conclusion, a large sample study of the reasonable dosage, safety and efficacy of N-acetylcysteine for bronchoscopic lavage therapy is urgently needed!

During mechanical ventilation, the patient developed persistent hypercarbonemia for more than 1 week, which was analyzed in 2 ways: ①. The main reason: the initial ventilator repeatedly indicated airway hypertension alarm and plenty of mucus could be seen under bronchoscopy. The primary consideration is related to the excessive mucus blockage in the airway and insufficient alveolar ventilation. ②. During subsequent mechanical ventilation, the patient was given a continuous lung-protective ventilation strategy to reduce ventilator-associated lung injury by giving a small tidal volume and ensuring a platform pressure of ≤30 cmH_2_O as much as possible, resulting in a “permissive hypercarbonemia” ventilation strategy. Permissive hypercarbonemia is defined as:^[17]^ in the treatment of respiratory diseases, a small tidal volume (6–8 ml/kg) and low minute ventilation are used to allow a slight increase in arterial blood carbon dioxide partial pressure (PaCO_2_ <80–100 mm Hg) and a certain degree of respiratory acidosis, to avoid pressure-volume injuries.^[18]^ Studies have shown that for ARDS, the efficacy was significantly better in the small tidal volume ventilation mild hypercarbonation group than in the high tidal volume ventilation group. Nevertheless, the risk of harm and death from persistent hypercarbonemia is also very high.^[19,20]^ Consequently, repeated bronchoscopic lavage in this patient relieved the mucus blockage in the patients airway and improved the hypercarbonemia caused by insufficient effective alveolar ventilation. The patient was considered for extracorporeal membrane oxygenation (ECMO) respiratory support during persistent hypercarbonemia according to the timing of ECMO initiation,^[21]^ but considering that the patients PaO_2_ was maintainable and the patient had a combined radical esophageal carcinoma, anastomotic-pulmonary fistula, and a poor prognosis, the PaCO_2_ showed a trend of gradual improvement after repeated bedside bronchoscopic lavage, and eventually, ECMO was not initiated.

Repeated sputum and bronchoscopic lavage cultures of Pseudomonas aeruginosa copper-green in this patient suggested clear secondary nosocomial infections, which is consistent with the conclusion of the clinical characteristics of the existing critically ill novel coronavirus pneumonia prone to secondary nosocomial infection.^[22]^ This patient had a history of esophageal cancer surgery, anastomotic-pleural fistula, and chronic obstructive pulmonary disease, combined with novel coronavirus pneumonia, secondary bronchial fistula, ARDS, complicated with hypoproteinemia, and multiple organ dysfunction. The disease itself suggested a low probability of cure. However, the patient was eventually cured and discharged and is currently surviving in good condition. The main experience of successful treatment is summarized as follows: ① Repeated bedside bronchoscopy with a large dose of 10 to 15 g/time of NAC nebulized inhalation solution lavage combined with routine nebulization and sputum suction airway management. ② Reasonable and invasive respiration support. ③ Reasonable anti-infective treatment. ④ Comprehensive nutritional support, immunotherapy, exceptional medical management and other comprehensive medical investment.

In addition, during the initial rescue and the first 2 bedside bronchoscope lavages, because the hospital does not have a positive pressure head mask, the doctor could only perform under the protection of protective clothing + goggles + face screen during the lavage process. From hospital admission to discharge, there was no case of medical staff infected with the novel coronavirus. It shows that for patients with novel coronavirus pneumonia, bedside bronchoscopy lavage may not necessarily require a positive pressure head mask. As long as the third level of protection is done, the infection can be avoided.

## Author contributions

**Data curation:** Meiffang Wang, Elaine Laihan Leung, Yan Liu, Guoshi Luo, Xin Qian, Chenglin Wu, Yijun Tang, Biyu Chen.

**Funding acquisition:** Meiffang Wang, Elaine Lai-Han Leung.

**Writing – original draft:** Yan Liu, Yizhong Zhang.

**Writing – review & editing:** Yijun Tang, Meiffang Wang, Elaine Laihan Leung.
